# Multiomics Machine Learning to Predict Neoadjuvant Chemotherapy Outcome and Relapse of Breast Cancer

**DOI:** 10.34133/bmef.0212

**Published:** 2026-01-27

**Authors:** Lili Wang, Xiaodong Zhang, Jing Zhang, Jian Liu, Ying Chen, Weiwei Huang, Xianhe Xie

**Affiliations:** ^1^ Department of Oncology, Molecular Oncology Research Institute, The First Affiliated Hospital, Fujian Medical University, Fuzhou 350000, China.; ^2^ Department of Oncology, National Regional Medical Center, Binhai Campus of The First Affiliated Hospital, Fujian Medical University, Fuzhou 350212, China.; ^3^ Department of Medical Oncology, Clinical Oncology School of Fujian Medical University, Fujian Cancer Hospital, Fuzhou 350014, China.; ^4^ Department of Radiology, Peking University First Hospital, Beijing 100034, China.; ^5^ Department of Pathology, Clinical Oncology School of Fujian Medical University, Fujian Cancer Hospital, Fuzhou 350014, China.; ^6^ Department of Radiology, Clinical Oncology School of Fujian Medical University, Fujian Cancer Hospital, Fuzhou 350014, China.; ^7^ Fujian Key Laboratory of Precision Medicine for Cancer, The First Affiliated Hospital, Fujian Medical University, Fuzhou 350000, China.

## Abstract

**Objective:** The aim of this study was to investigate multiomics (MO) integration with stacked-ensemble learning for predicting neoadjuvant chemotherapy (NAC) response and recurrence risk in breast cancer (BC). **Impact Statement:** This study demonstrates that a stacked-ensemble learning model integrating clinicopathologic and magnetic resonance imaging (MRI)-based intratumoral heterogeneity biomarkers effectively predicts NAC response and postoperative recurrence risk in BC patients. These findings underscore MO and machine learning’s potential to optimize clinical decision-making. **Introduction:** Selecting BC patients who will benefit from NAC remains challenging. **Methods:** We retrospectively analyzed 124 BC patients receiving NAC (3 to 8 cycles) prior to mastectomy. Two radiomics signatures—RadS_ET_ and RadS_ITH_—were derived from pre-NAC high-resolution dynamic MRI to track entire-tumor and intratumoral heterogeneous characteristics, respectively. These signatures were integrated with clinicopathologic indicators using stacked-ensemble learning algorithms to predict pathological complete response (pCR) and 3-year disease-free survival (DFS). **Results:** Among the 124 patients, the pCR rate was 26.6%. For pCR prediction, RadS_ITH_ and RadS_ET_ yielded areas under the curve (AUCs) of 0.798 and 0.770, respectively. The MO-integrated model, combining RadS_ITH_, RadS_ET_, clinical N stage, and molecular subtype, achieved a significantly higher AUC (0.917; 95% confidence interval [CI], 0.860 to 0.958; *P* < 0.05) than individual models. Postoperative recurrence occurred in 13.6% of patients. The elastic-net Cox model achieved a DFS concordance index of 0.78 (95% CI, 0.72 to 0.83) using pre-NAC variables (MO-predicted pCR, Response Evaluation Criteria in Solid Tumors response, RadS_ITH_), and 0.81 (95% CI, 0.76 to 0.92) with post-NAC variables (pathologic grade, pCR status, pT stage, and pN stage). **Conclusion:** The MO integration with stacked-ensemble learning effectively predicts NAC response and recurrence risk in BC.

## Introduction

Breast cancer (BC) is the most prevalent malignant tumor in women and has the highest incidence rate among newly diagnosed cancers globally [1,2]. Neoadjuvant chemotherapy (NAC) is the standard treatment for locally advanced BC, aiming to reduce tumor burden and increase eligibility for breast-conserving surgery [3,4].

Achieving a pathological complete response (pCR) following NAC, particularly in patients with triple-negative BC (TNBC) or human epidermal growth factor receptor 2 (HER2)-positive subtypes, is strongly associated with markedly improved overall survival and disease-free survival (DFS) [5–8]. However, not all patients benefit from NAC, and therapeutic responses exhibit considerable inter-patient variability [9]. Additional therapies impose a substantial economic burden on these NAC nonresponders because of extending treatment cycles, and may potentially delay the effective therapeutic course [10]. Therefore, accurately identifying BC patients unlikely to respond to NAC is crucial for enabling individualized treatment strategies and improving cure rates.

BC, especially in its locally advanced stages, is characterized by high intratumoral heterogeneity (ITH) [11]. ITH arises from the accumulation of diverse molecular and genetic alterations in tumor cell subpopulations during growth, leading to genetic variation and phenotypic differences across tumor subregions [12]. In solid tumors, ITH is a key driver of adaptive resistance to chemotherapy, closely linked to disease progression and reduced survival [13,14]. It may also underlie the observed variations in NAC response among patients with similar clinical profiles [15]. Consequently, quantitative assessment of ITH holds promises as a valuable biomarker for predicting clinical outcomes [16].

Noninvasive imaging technologies, particularly magnetic resonance imaging (MRI), represent state-of-the-art tools for BC detection, diagnosis, and treatment evaluation. The National Comprehensive Cancer Network Clinical Practice Guidelines in Oncology for Breast Cancer (version 4; 2023) specifically recommend MRI for post-NAC response assessment [17]. However, research on the predictive utility of MRI prior to NAC initiation remains limited. Radiomics, the extraction of quantitative features from medical images, provides valuable supplementary information that enhances BC diagnosis, risk stratification, treatment planning, and prognostic prediction [18–20]. While conventional radiomics analyzes the entire tumor volume, capturing overall phenotypic characteristics and partially reflecting ITH, it often overlooks regional phenotypic variations due to feature averaging [21]. To address this, subregional radiomics analysis has emerged. For instance, Hu et al. [22] pioneered this approach in esophageal cancer, demonstrating its ability to predict survival outcomes after chemoradiotherapy by effectively quantifying tumor subregions linked to growth or invasion and rigorously evaluating the impact of ITH on radiomic analysis. Furthermore, emerging technologies like magnetic resonance endoscopy and optical coherence tomography offer notable potential for noninvasive, real-time ITH analysis and therapeutic response monitoring, leveraging their micrometer-scale spatial resolution [23,24].

Therefore, this study aims to develop and validate a stacked-ensemble machine learning model integrating multiomics (MO) data—specifically MRI-based ITH measures, clinical factors, and pathological indicators—to effectively predict both pCR and recurrence risk in BC patients before NAC initiation.

## Results

### Baseline patient characteristics

Among the enrolled 124 BC patients, 33 (26.6%) achieved pCR following NAC, while 91 (73.4%) did not achieve pCR. Patient demographic and baseline characteristics are summarized in Table 1.

**Table 1.** Demographic and baseline characteristics of patients. Data are presented as mean (standard deviation) or number of observations.

Characteristics	Treatment response (*n* = 124)		
	pCR (*n* = 33)	Non-pCR (*n* = 91)	*P* value
Age (years)	45.8 (10.6)	47.4 (9.1)	0.13
Menstrual status			0.80
Premenopausal (*n* = 69)	19	50	
Postmenopausal (*n* = 55)	14	41	
Baseline clinical T stage			0.34
Clinical T1–2 (*n* = 74)	22	52	
Clinical T3–4 (*n* = 50)	11	39	
Baseline clinical N stage			0.23
Clinical N0 (*n* = 28)	11	17	
Clinical N1 (*n* = 57)	13	44	
Clinical N2–3 (*n* = 39)	9	30	
Baseline clinical AJCC stage			0.09
Stage I–II (*n* = 56)	19	37	
Stage III (*n* = 68)	14	54	
HR status			0.63
Positive (*n* = 72)	18	54	
Negative (*n* = 52)	15	37	
HER2 status			<0.001 ^a^
Negative (*n* = 74)	11	63	
Positive (*n* = 50)	22	28	
Ki-67 status			0.01 ^a^
Low proliferation (<30%) (*n* = 27)	2	25	
High proliferation (≥30%) (*n* = 97)	31	66	
Molecular subtype			<0.001 ^a^
HR positive, HER2 negative (luminal) (*n* = 45)	4	41	
HR positive, HER2 positive (TPBC) (*n* = 27)	14	13	
HR negative, HER2 positive (HER2 positive) (*n* = 23)	8	15	
HR negative, HER2 negative (TNBC) (*n* = 29)	7	22	
Cycles of NAC treatment			0.55
≥6 cycles (*n* = 89)	25	64	
<6 cycles (*n* = 35)	8	27	
NAC regimens			
Anthracyclines (*n* = 83)	12	71	0.003 ^a^
Taxanes (*n* = 117)	33	84	0.15
Platinum agents (*n* = 31)	16	15	0.16
Biopsy p-Grade			0.48
bG1 (*n* = 0)	0	0	
bG2 (*n* = 84)	24	60	
bG3 (*n* = 40)	9	31	
Post-NAC RECIST status			0.02 ^a^
Complete response (*n* = 0)	0	0	
Partial response (*n* = 105)	33	72	
Stable disease (*n* = 14)	0	14	
Progressive disease (*n* = 5)	0	5	
Postoperative pathologic grade			<0.001 ^a^
Negative and unavailable (*n* = 40)	33	7	
pG1 (*n* = 0)	0	0	
pG2 (*n* = 56)	0	56	
pG3 (*n* = 28)	0	28	
pTNM stage			<0.001 ^a^
Stage 0 (*n* = 28)	28	0	
Stage I (*n* = 24)	0	24	
Stage II (*n* = 45)	5	40	
Stage III (*n* = 27)	0	27	
pT stage			<0.001 ^a^
ypT0 (*n* = 33)	33	0	
ypT1–2 (*n* = 86)	0	86	
ypT3–4 (*n* = 5)	0	5	
pN stage			<0.001 ^a^
pN0 (*n* = 63)	28	35	
pN1 (*n* = 35)	5	30	
pN2 (*n* = 16)	0	16	
pN3 (*n* = 10)	0	10	

AJCC, American Joint Committee on Cancer; bG, biopsy grade; HER2, human epidermal growth factor receptor 2; HR, hormone receptor; N, node; NAC, neoadjuvant chemotherapy; p-Grade, pathological grade; pCR, pathological complete response; pG, pathological grade; pN, pathological node; pT, pathological tumor; pTNM, pathological tumor-node-metastasis; RECIST, Response Evaluation Criteria in Solid Tumors; T, tumor; TNBC, triple-negative breast cancer; TPBC, triple-positive breast cancer

^a^ Significant based on the chi-square test.

### Radiomics analysis

**Fig. 1.**
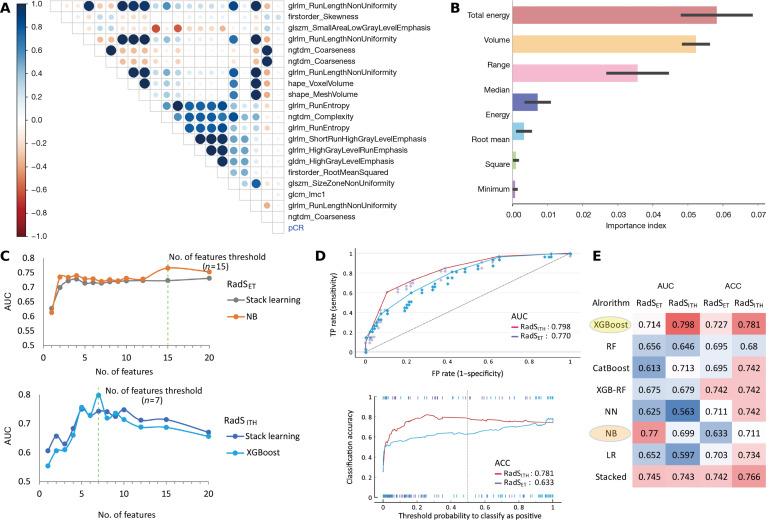
Feature selection and model selection for the construction of RadS_ET_ and RadS_ITH_. (A) Hierarchical cluster analysis heatmap of the RadS_ET_ features and their association with pCR. (B) Importance ranking of the RadS_ITH_ features based on the SHAP plot. (C) Flowchart of the stepwise feature selection procedure for RadS_ET_ and RadS_ITH_ construction, wherein features were sequentially added to maximize the model’s AUC. (D) Comparison of AUC and ACC plots between RadS_ET_ (constructed using the NB learner) and RadS_ITH_ (constructed using the XGBoost learner) based on the selected candidate models. (E) AUC and ACC results for the 8 machine learners for model selection. ACC, accuracy; AUC, area under the curve; FP, false positive; LR, logistic regression; NB, Naïve Bayes; NN, neural network; pCR, pathological complete response; RadS_ET_, radiomics signature using entire tumor; RadS_ITH_, radiomics signature using intratumoral heterogeneity; RF, random forest; SHAP, SHapley Additive exPlanations; TP, true positive; XGB-RF, XGBoost with RF; XGBoost, extreme gradient boosting.

The results of the correlation analysis between the RadS_ET_ and RadS_ITH_ features and pCR are exhibited in Fig. 1A and B, respectively. Based on stepwise feature selection analysis, a total of 15 RadS_ET_ features and 7 RadS_ITH_ features with the highest area under the curve (AUC) values were selected as candidate features (Fig. 1C). Among the 8 evaluated machine learning algorithms, extreme gradient boosting (XGBoost) demonstrated the highest performance and was selected as the optimal learner (leading learner) for constructing the RadS_ITH_. This model achieved an AUC of 0.798 (95% CI: 0.709 to 0.857) and an overall accuracy of 78.1% for pCR prediction. Similarly, Naïve Bayes (NB) was selected as the optimal learner for constructing the RadS_ET_, which achieved an AUC of 0.770 (95% CI: 0.689 to 0.832) and an overall accuracy of 63.3% (Fig. 1D). The AUC and accuracy results for all 8 machine learning algorithms evaluated during model selection for both RadS_ET_ and RadS_ITH_ are summarized in Fig. 1E.

### MO analysis for pCR prediction

**Fig. 2.**
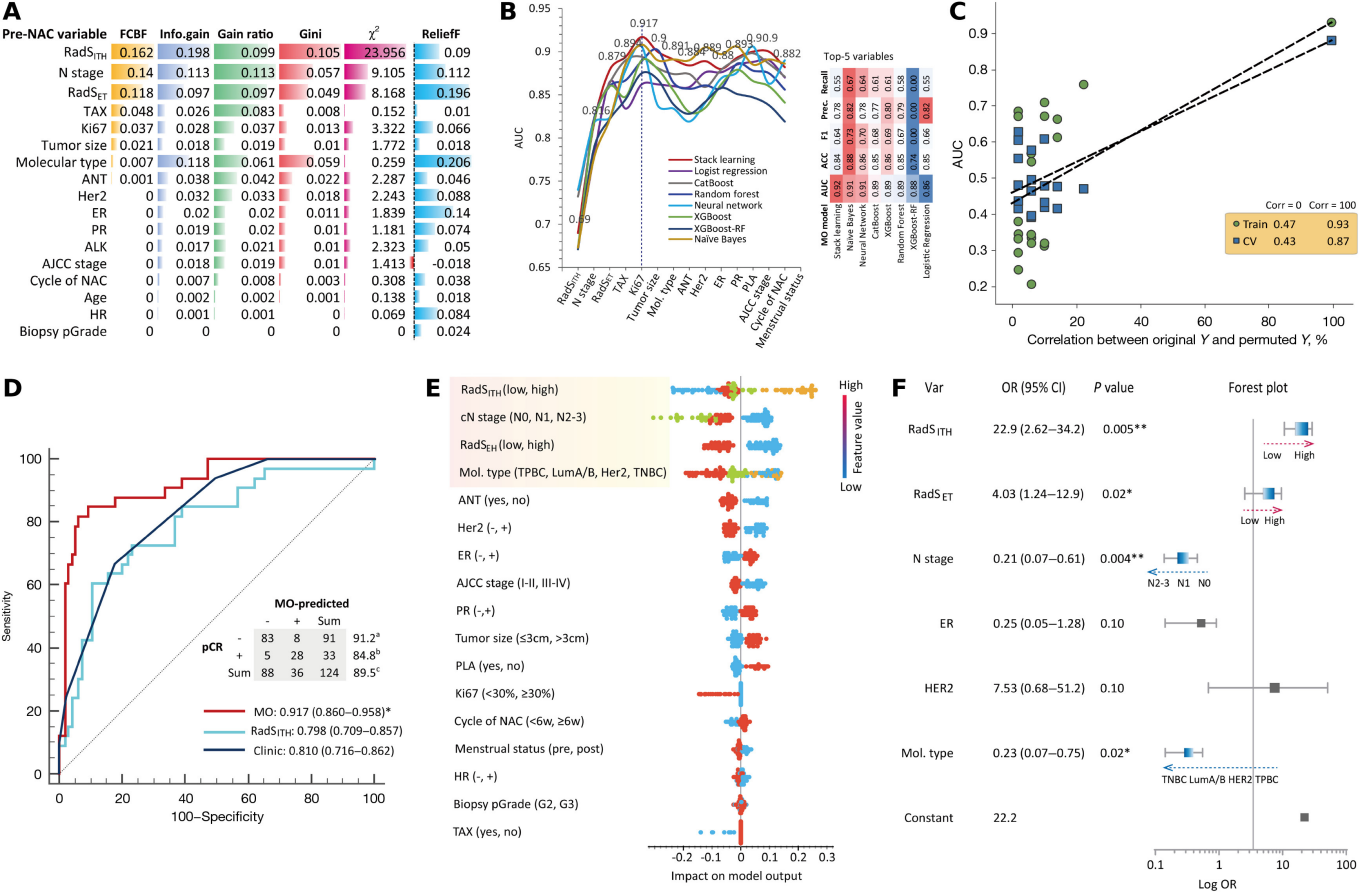
MO integration with stacked machine learning algorithms for pCR. (A) Importance ranking of integrated MO features based on a correlation analysis with the dedicated algorithms. (B) Stepwise feature selection and model selection for the MO-integrated model, in which integrating the top 5 factors via stacked learning achieved the highest AUC for pCR prediction. (C) Overfitting analysis of the selected MO model with a permutation plot, demonstrating minimal error between the training and cross-validation test sets (0.93/0.87). (D) Comparison of the ROC curve analyses of the MO model, a clinic model (clinical + pathological variables), and RadS_ITH_. (E) Explanation of the MO model using the SHAP plot. Notably, RadS_ITH_ had the greatest effect on the prediction of pCR. (F) A similar result was observed in the multivariable LR analysis, in which RadS_ITH_ was found to be an independent predictor of pCR. ^a^ Specificity; ^b^ sensitivity; ^c^ accuracy. *P < 0.05. **P < 0.01. AJCC, American Joint Committee on Cancer; ANT, anthracycline; AUC, area under the curve; CI, confidence interval; cN, clinical node; CV, cross-validation; ER, estrogen receptor; FCBF, fast correlation-based feature; HER2, human epidermal growth factor receptor 2; LR, logistic regression; MO, multiomics; N, node; NAC, neoadjuvant chemotherapy; OR, odds ratio; p-Grade, pathological grade; pCR, pathological complete response; PLA, platinum agent; PR, progesterone receptor; RadS_ET_, radiomics signature using entire tumor; RadS_ITH_, radiomics signature using intratumoral heterogeneity; ROC, receiver operating characteristic; TAX, taxane; TNBC, triple-negative breast cancer; TPBC, triple-positive breast cancer.

The newly calculated RadS_ET_ and RadS_ITH_ scores were integrated with 15 pre-NAC clinicopathologic variables into an MO model for pCR prediction. First, stepwise feature selection analysis ranked the importance of the 17 MO features associated with pCR (Fig. 2A). Second, the optimal MO model was selected by iteratively adding the ranked features to 8 competitive learners in descending order of importance until peak performance was achieved. RadS_ITH_, clinical N stage, RadS_ET_, and molecular BC subtype emerged as the top 4 predictors of pCR. The MO model incorporating these 4 features using stacked-ensemble learning achieved the highest performance (AUC: 0.917), followed by NB (AUC: 0.908), neural networks (NNs) (AUC: 0.906), gradient boosting with categorical features support (CatBoost) (AUC: 0.894), XGBoost (AUC: 0.890), random forest (RF) (AUC: 0.889), XGBoost-RF (AUC: 0.875), and logistic regression (LR) (AUC: 0.862) (Fig. 2B).

**Fig. 3.**
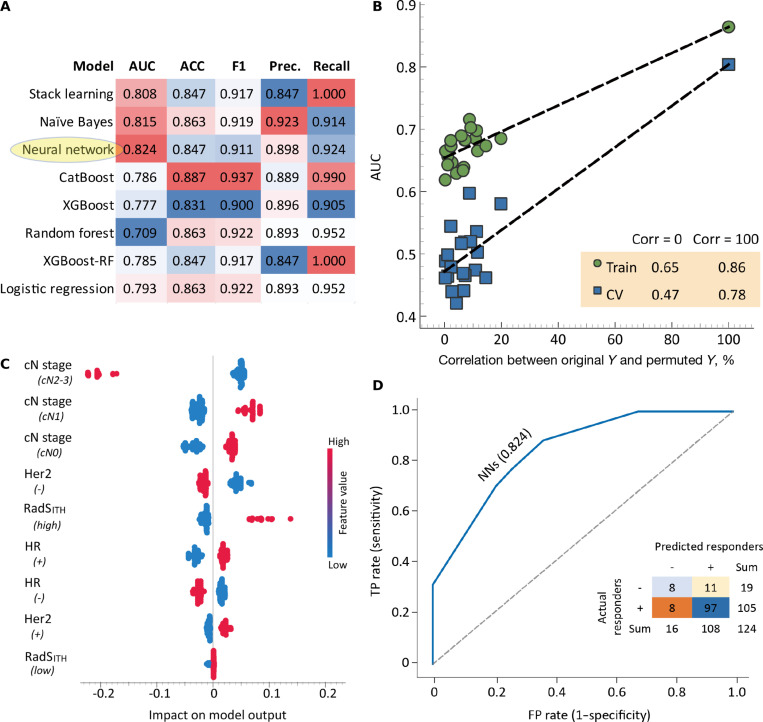
Performance of MO integration for NAC responders. (A) Model selection results; the NN model that included the 4 leading features (i.e., clinical N stage, RadS_ITH_, HER2, and HR) had the highest AUC, and was selected as the candidate model for discrimination between responders and nonresponders. (B) Permutation test of the NN model for overfitting evaluation. (C) SHAP plot showing the importance of features selected at the candidate NN model. (D) ROC curve analysis and the matrix plot of the NN model for predictive performance. ACC, accuracy; AUC, area under the curve; cN, clinical node; CV, cross-validation; FP, false positive; HER2, human epidermal growth factor receptor 2; HR, hormone receptor; MO, multiomics; N, node; NN, neural network; Prec., precision; RadS_ITH_, radiomics signature using intratumoral heterogeneity; RF, random forest; ROC, receiver operating characteristic; SHAP, SHapley Additive exPlanations; TP, true positive; XGBoost, extreme gradient boosting.

Next, the potential overfitting of the optimal MO model was assessed using permutation testing (Fisher’s permutation test). Minimal error was observed between the training and cross-validation results, indicating good generalizability (Fig. 3C). To evaluate its performance advantage, the stacked-learning MO model was compared with the RadS_ITH_ model and clinical model based on pre-NAC clinicopathologic variables (Fig. 2D). Pairwise receiver operating characteristic (ROC) curve analysis revealed that the MO model achieved a significantly higher AUC (0.917; 95% CI: 0.860 to 0.958) than both the RadS_ITH_ model (0.798; 95% CI: 0.709 to 0.857; *P* = 0.004) and the clinical model (0.810; 95% CI: 0.716 to 0.862; *P* = 0.007). At the optimal threshold (maximizing Youden’s index), the MO model demonstrated a sensitivity of 84.8%, a specificity of 91.2%, and an overall accuracy of 89.5% for pCR prediction. No significant AUC difference was found between the RadS_ITH_ and clinical models (*P* = 0.83).

To enhance model explainability, the contributions of features within the stacked-learning MO model were interpreted and visualized using SHAP (SHapley Additive exPlanations) analysis (Fig. 2E). Similarly, multivariate LR analysis confirmed RadS_ITH_ (odds ratio [OR] = 22.9; 95% CI: 2.62 to 34.2; *P* = 0.005), clinical N stage (OR = 0.21; 95% CI: 0.07 to 0.61; *P* = 0.004), RadS_ET_ (OR = 4.03; 95% CI: 1.24 to 12.9; *P* = 0.02), and molecular classification (OR = 0.23; 95% CI: 0.07 to 0.75; *P* = 0.02) as 4 independent predictors of pCR (Fig. 2F).

The performance of the MO model in predicting Response Evaluation Criteria in Solid Tumors (RECIST) response status is summarized in Fig. 3. During the stepwise selection process, NN-based framework incorporating 4 key features, clinical N stage, RadS_ITH_, HER2, and HR status, achieved the best performance (AUC: 0.824; 95% CI: 0.768 to 0.887) in discriminating responders from nonresponders. Its high sensitivity enabled the identification of 92.4% (97/105) of patients who responded to NAC.

### Association of MO with postoperative recurrence

Among the 124 patients, 110 completed 3 years of effective follow-up. Of these, 15 patients (13.6%) experienced postoperative recurrence within the 3-year period.

**Fig. 4.**
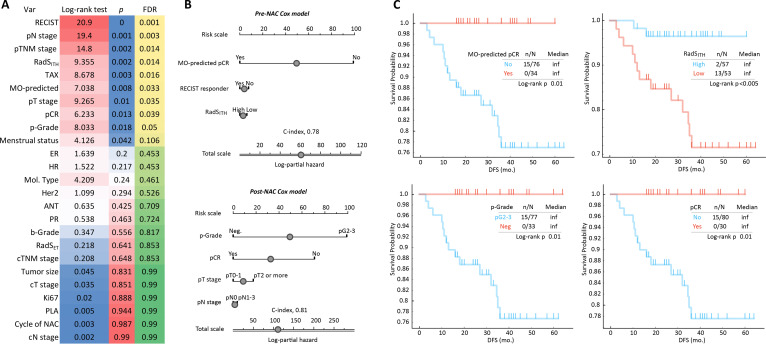
The predictive ability of MO for the postoperative recurrence. (A) Univariate log-rank and FDR test examining the associations between the MO factors and postoperative recurrence. (B) Two explainable nomograms were constructed using pre-NAC and post-NAC factors, respectively, based on the elastic-net Cox regression analysis to calculate the individual risk of postoperative recurrence. (C) Kaplan–Meier plots of patient groups stratified by risk factors with DFS. ANT, anthracycline; C-index, concordance index; cN, clinical node; cT, clinical tumor; cTNM, clinical tumor-node-metastasis; DFS, disease-free survival; ER, estrogen receptor; FDR, false discovery rate; HER2, human epidermal growth factor receptor 2; HR, hormone receptor; Inf, infinity; MO, multiomics; NAC, neoadjuvant chemotherapy; p-Grade, pathological grade; pCR, pathological complete response; pG, pathological grade; PLA, platinum agent; pN, pathological node; PR, progesterone receptor; pT, pathological tumor; pTNM, pathological tumor-node-metastasis; RadS_ET_, radiomics signature using entire tumor; RadS_ITH_, radiomics signature using intratumoral heterogeneity; RECIST, Response Evaluation Criteria in Solid Tumors; TAX, taxane.

Univariate log-rank analysis identified 10 clinic–radiomic–pathological variables significantly associated (*P* < 0.05) with postoperative recurrence (Fig. 4A). The Cox Elastic-net regression model identified 3 significant pre-NAC predictors of recurrence: MO-predicted pCR status, RECIST response status, and RadS_ITH_. This model achieved a concordance index (C-index) of 0.78 (95% CI: 0.72 to 0.83) for predicting recurrence. Among post-NAC variables, the final regression model for predicting recurrence (C-index: 0.81; 95% CI: 0.76 to 0.92) identified significant predictors as p-Grade, pCR status, pT stage, and pN stage (Fig. 4B). Kaplan–Meier survival curves were generated for the top predictors from each model. Significant survival differences were observed between the stratified patient groups defined by these predictors (Fig. 4C).

## Discussion and conclusion

The pretreatment assessment of NAC response and long-term prognostic outcomes in BC patients is crucial for guiding treatment decisions [25]. In this study, we performed a stepwise analysis of high-throughput MRI radiomic features and developed 2 imaging signatures (RadS_ET_ and RadS_ITH_) strongly associated with NAC response. Our results indicate that MRI features, particularly RadS_ITH_ derived from ITH analysis, represent potential pretreatment biomarkers for predicting pCR in BC patients undergoing NAC. Furthermore, integrating these imaging biomarkers with clinicopathological variables into a hybrid MO model by a novel stacked-ensemble machine learning algorithm significantly improved pCR predictive performance. Importantly, this novel MO biomarker also demonstrated an association with postoperative recurrence risk, suggesting its potential utility in postoperative prognostic assessment. Collectively, our findings offer a new approach for personalized NAC efficacy prediction in BC, which may aid in optimizing treatment plans and improving patient outcomes.

The pCR rates vary with clinicopathological status. Consistent with prior studies [26–28], which identified associations between higher pCR rates and factors like high tumor grade, HER2-positive status, elevated Ki-67 index, and ER-negative status, our study confirmed a significant association between pre-NAC HER2 status and pCR. We also observed elevated pCR rates in HER2-positive and triple-positive BC (TPBC) subtypes compared to others.

This study established 2 independent imaging signatures (RadS_ET_ and RadS_ITH_) and an MO signature for pCR prediction. Aligning with previous research [29,30], our results underscore the importance of MO integration for NAC response assessment. Employing stepwise feature and model selection, we ranked 17 MO factors related to pCR and established an optimal model. While stacked-ensemble learning has been previously applied to pCR prediction [29,31], our approach uniquely incorporates subregional tumor heterogeneity (RadS_ITH_), enhancing both predictive accuracy and clinical interpretability. RadS_ITH_, clinical N stage, RadS_ET_, and BC molecular classification emerged as the top 4 predictors of pCR, corroborating the known importance of molecular subtype and clinical N stage [32,33]. In addition, compared with the state-of-the-art I-SPY TRIAL models with various algorithms such as conventional morphology, radiomics, and deep learning for the prediction of pCR [34], our RadS_ITH_ revealed comparable performance (0.798). Furthermore, an MO-integrated model, by combining preoperative MO factors such as RadS_ITH_, clinical, and molecular factors, did achieve notably higher AUC (0.917) and thereby improved the prediction performance for pCR.

Regarding model development, we utilized novel stacked learning, an ensemble method that selects the best-performing model from multiple base learners to improve predictive accuracy. Although less common than other methods, Mahajan et al. [33] demonstrated its potential for performance enhancement. Furthermore, we assessed potential overfitting in our limited sample using permutation testing, revealing minimal error between training and cross-validation sets. This strategy aligns with recommended approaches for mitigating overfitting in predictive analytics for BC, particularly in ROC curve estimation [35]. The superior AUC of our stacked-learning MO model compared to both the stand-alone RadS_ITH_ model and a non-RadS_ITH_ model (based solely on preoperative clinicopathological variables) is consistent with findings from multimodal deep learning models combining clinical data with preprocessed MRI images [36]. Such multimodal fusion approaches integrating clinical and imaging data consistently outperform models relying solely on clinical information [36]. Finally, to enhance model interpretability, we employed SHAP plots to visualize feature contributions within the MO model. This technique, distinct from conventional methods like LR analysis, provides a more intuitive model explanation [37]. Our approach resonates with the emphasis on interpretability seen in novel frameworks like the self-interpretable deep-learning network (SIDLN) proposed by Gao et al. [38], highlighting its capacity for both quantitative and qualitative explanation of feature importance.

Additionally, we evaluated the performance of MO integration in predicting NAC clinical efficacy (RECIST response). Four MO features—clinical N stage, RadS_ITH_, HER2 status, and HR status—were identified as associated with treatment response. Integrating these features within an NN model yielded optimal predictive performance (AUC: 0.824). Clinically, such models could effectively differentiate NAC responders from nonresponders, enabling clinicians to tailor treatment plans, reduce ineffective therapies, and improve outcomes.

Our model also effectively predicted postoperative recurrence risk using pre- and posttreatment MO variables, potentially informing long-term follow-up strategies. Kaplan–Meier analysis confirmed significant survival differences based on these predictions. Key predictors of recurrence risk included MO-predicted pCR status, RECIST response status, RadS_ITH_, p-Grade, pCR status, pT stage, and pN stage. These findings highlight the potential of MO features for recurrence prediction and support prior research [39], demonstrating that MO data integration enhances BC recurrence prediction accuracy.

Nonetheless, this study has limitations, including a relatively small, single-center cohort and its retrospective design. These factors might potentially influence the broader applicability of the results, as the patient cohort from a single institution may not encompass the full spectrum of demographic and clinical characteristics encountered in wider practice. In order to minimize these effects, several critical strategies, such as feature and model selection and 5-fold cross-validation with permutation test, were adopted to reduce the overfitting risk. Multicenter prospective validations are still essential, and further studies are needed to confirm generalizability and clinical applicability.

In this real-world retrospective study, we employed MO data integration and stacked-ensemble learning to predict NAC response and recurrence risk in BC patients. Our findings not only provide a novel approach for predicting treatment response and recurrence risk but also identify potential biomarkers to advance personalized medicine strategies in BC management.

## Methods

### Patients

This retrospective study analyzed data from BC patients treated at Fujian Cancer Hospital between April 2017 and May 2023. The research protocol was approved by the local institutional ethics review committee (K2025-020-01). In accordance with the retrospective nature of the study and the anonymization of patient data, the requirement for written informed consent was waived. All research procedures adhered to the principles of the Declaration of Helsinki (1964) and its subsequent amendments.

The primary study cohort comprised patients with clinical records and imaging data available from Fujian Cancer Hospital. Inclusion criteria were as follows: (a) Initial diagnosis of locally advanced BC; (b) no prior surgery, radiotherapy, or chemotherapy for BC; (c) absence of distant metastasis at diagnosis; (d) completion of standard NAC followed by radical mastectomy; (e) pre-NAC breast MRI scan performed within 2 weeks prior to NAC initiation; and (f) availability of comprehensive clinical, imaging, and pathological data meeting the study criteria.

**Fig. 5.**
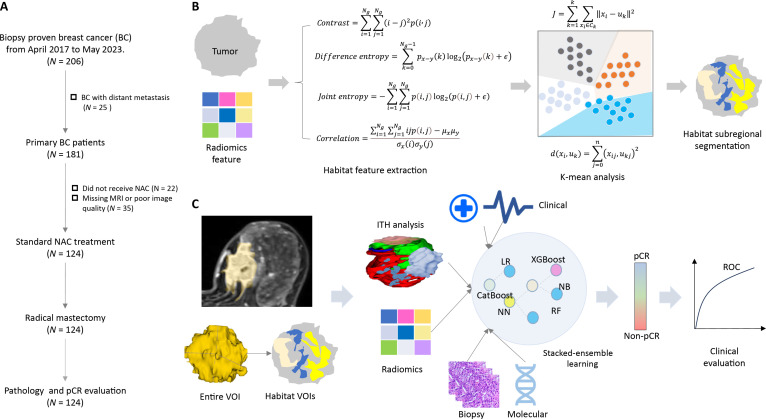
Flowchart of MO analysis for the prediction of pCR in BC patients who receive NAC. (A) Procedure of patient inclusion and exclusion criteria. (B) Metrics for radiomics feature extraction, and habitat RadS_ITH_ analysis. (C) Metrics for MO integration and clinical evaluation. BC, breast cancer; ITH, intratumoral heterogeneity; LR, logistic regression; MO, multiomics; MRI, magnetic resonance imaging; NAC, neoadjuvant chemotherapy; NB, Naïve Bayes; NN, neural network; pCR, pathological complete response; RadS_ITH_, radiomics signature using intratumoral heterogeneity; RF, random forest; ROC, receiver operating characteristic; VOI, volume of interest; XGBoost, extreme gradient boosting.

Initially, 206 patients were identified as potentially eligible. Of these, 82 were excluded: 25 due to distant metastasis, 22 for not receiving NAC therapy, and 35 due to missing imaging sequences or poor image quality precluding analysis. Consequently, 124 patients with complete datasets who underwent standard NAC followed by surgery were included in the final analysis. The patient selection process, including inclusion/exclusion criteria and study flow, is presented in Fig. 5A.

### Patient management

Patients received a standard NAC regimen consisting of taxanes, platinum agents, and anthracyclines, with or without HER2-targeted therapy based on molecular subtype. Most patients received 6 to 8 cycles of NAC; however, 6 patients received only 2 to 3 cycles due to insufficient tumor regression or patient preference for proceeding to surgery. Following NAC completion, all patients underwent surgical resection of breast tumors and regional lymph nodes. The resected tissue specimens were then processed for histopathological evaluation, including fixation, paraffin embedding, sectioning, and hematoxylin and eosin staining.

### Pathological assessment and biomarker definition

Diagnoses for all included patients were confirmed by core needle biopsy and subsequent pathological assessment. Two pathologists, each with over 10 years of specialized experience in breast pathology, independently reviewed all biopsy and surgical resection specimens.

Immunohistochemistry (IHC) was performed to assess the expression levels of estrogen receptor (ER), progesterone receptor (PR), HER2, and the Ki-67 proliferation index. ER and PR are collectively termed hormone receptors (HRs). HR-positive status was defined as nuclear staining in ≥1% of tumor cells for either ER or PR. Conversely, HR-negative status required nuclear staining in <1% of tumor cells for both ER and PR. Tumors with HER2 IHC membrane staining scored as 3+ were classified as HER2-positive. Cases scored as 2+ underwent fluorescence in situ hybridization to confirm HER2 gene amplification. Ki-67 positivity was defined as nuclear staining in ≥30% of tumor cells. Additionally, the pathological grade (p-Grade) and pathological stage (according to the American Joint Committee on Cancer [AJCC] 8th edition criteria) were evaluated. In this study, pCR in the breast was defined as the absence of invasive residual tumor (ypT0/Tis).

### Multiparametric MRI protocol

All breast MRI examinations were performed on a 3.0-T system (Discovery MR750w, GE Healthcare) equipped with an 8-channel breast coil. All images were acquired via bilateral axial views. The routine protocol included turbo spin echo T1-weighted imaging (repetition time [TR]/echo time [TE], 4.5 ms/2.3 ms; field of view [FOV], 36 × 36 cm; slice thickness, 3 mm) and T2-weighted fat-suppressed spin echo (TR/TE, 3,600 ms/1,020 ms; FOV, 36 × 36 cm; slice thickness, 5 mm). Echo planar diffusion-weighted imaging was performed before contrast enhancement (TR/TE, 2,600 ms/78 ms; FOV, 36 × 36 cm; slice thickness, 5 mm), with *b*-values of 0 and 800 s/mm^2^. Dynamic contrast-enhanced (DCE) MRI used a T1-weighted gradient echo sequence (TR/TE, 7.8 ms/4.3 ms; flip angle, 12°; FOV, 36 × 36 cm; slice thickness, 1 mm). Image subtraction was performed after all contrast phases. Gadolinium-based contrast agent was administered at a dose of 0.1 mmol/kg, using a power injector compatible with magnetic resonance at a rate of 3 ml/s, followed by a flush with 20 ml of physiological saline.

### Image preprocessing

Entire tumor segmentation was performed using an in-house software (Multilabel Tool, version 1.5; Normal Intelligence Medical Technology Co., Ltd., Shanghai, China) provided by the Key Laboratory of MRI at East China Normal University, Shanghai. Two radiologist readers with 5 and 10 years of experience, respectively, in breast MRI manually delineated the entire tumor volume of interest (VOI) on DCE arterial phase images. The agreement of segmentation between the 2 readers arrived at a median Dice score of 95% was initially tested in 20 cases to ensure reproducibility in imaging annotation. This delineation integrated information from clinical findings and core biopsy reports. For patients presenting multiple lesions, the largest tumor was selected as the primary lesion of interest.

Subsequently, to mitigate intensity variations between different cases, the segmented images underwent normalization. Grayscale values within the image data were normalized using standard scores (*z* scores), with a mean (*μ*) of 0 and a standard deviation (*σ*) of 1. This was computed using *μ* and *σ* of the grayscale values within the VOI. To mitigate the influence of outliers, the intensity values were clipped to the range of *μ* ± 3*σ* prior to normalization, and histograms were subsequently adjusted to approximate a standard normal distribution.

Additionally, to minimize boundary inaccuracies potentially introduced during manual delineation and ensure comprehensive coverage of all tumor tissue, the original tumor VOI contour boundary was first expanded outward by 3 mm. This expanded boundary was then subjected to erosion processing.

### Habitat subregion generation

To identify intratumoral subregions, 5 texture parameters derived from the gray-level co-occurrence matrix (GLCM) were utilized: contrast, difference entropy, joint energy, joint entropy, and correlation. These parameters were applied pixel-wise across the entire tumor VOI to generate feature maps, enabling the identification of distinct habitat subregions. Based on the local texture characteristics of each pixel, the tumor region was segmented into clusters. The optimal number of clusters was determined to be 3, as this configuration yielded the highest stability and reproducibility at the Calinski–Harabasz value selection analysis [40]. Each resulting cluster was assigned a unique color label, and the resulting cluster label map visually depicts the spatial distribution of the intratumoral habitat subregions. The detailed methodology for generating these habitat subregions is illustrated in Fig. 1B.

### Radiomics feature extraction

Radiomics features were extracted from the entire VOI and the defined habitat subregions of the tumor on the normalized arterial and venous phase DCE images using the Pyradiomics package (version 2.7.7; Python 3.7). During feature extraction, the following preprocessing steps were applied: voxel spacing was resampled to 3 mm, a bin width of 5 was used for discretization, and the normalization scale was set to 1,000. To capture both original and derived image characteristics, 8 image filters were applied: Wavelet, Laplacian of Gaussian, Gradient, Local Binary Pattern in 3 Dimensions, Exponential, Square, Square Root, and Logarithm. A total of 1,558 features were extracted, categorized as follows: 306 first-order features, 14 shape features, 374 GLCM features, 272 gray-level size zone matrix features, 272 gray-level run length matrix features, 82 neighboring gray-tone difference matrix features, and 238 gray-level dependence matrix features. A detailed description of all extracted radiomics features is provided in the Supplementary Materials (Text S1).

### Feature selection and model construction

To select informative features while mitigating bias and overfitting risk, a 2-step dimensionality reduction strategy was employed: First, features exhibiting high redundancy were identified and removed. This involved calculating the pairwise Pearson correlation coefficient (PCC) between all features. Additionally, the false discovery rate (FDR) was assessed using the Mann–Whitney *U* test. Features with an absolute PCC value greater than 0.85 (i.e., |PCC| > 0.85) and an FDR-adjusted *P* value greater than 0.05 were excluded. Second, 6 distinct feature selection algorithms—Information Gain, Gain Ratio, Gini Index, χ^2^ (chi-square), ReliefF, and Fast Correlation-Based Filter (FCBF)—were applied to evaluate and rank the relevance of the remaining features to the target variable. To enhance the stability of the selection process and minimize the impact of randomness inherent in individual algorithms, only features consistently ranked within the top 5% across all 6 algorithms plus the initial statistical filter (totaling 7 selection methods) were retained for final model construction.

Next, to construct the radiomics signatures, we first developed an entire volumetric radiomics signature (RadS_ET_) based on features extracted from the entire tumor volume. Additionally, to quantify ITH, we constructed a habitat radiomics signature (RadS_ITH_) incorporating features derived from the identified intratumoral habitat subregions. Selecting the optimal machine learning algorithm for real-world radiomics analysis is challenging due to inherent data heterogeneity, population variations, and specific learning tasks. To address this challenge, we implemented a diverse set of 7 popular baseline machine learning algorithms and employed an automated stacking ensemble framework to identify their optimal combination. The initial layer of the stacking framework comprised 7 baseline learners: LR, NNs, NB, RF, XGBoost, XGBoost with RF (XGB-RF), and CatBoost. The outputs from the 7 base learners were combined to create a new feature set, which serves as input to a meta-model for the final ensemble prediction. Therefore, our stacking model is an ensemble method that computes a meta model from several base models. If no aggregation input is given, the default base models are used.

### Model optimization and validation

A random grid search strategy was used to optimize both the hyperparameters of the models and the configurations of the radiomics features utilized. The optimal features and parameters were selected based on minimizing the log-loss metric evaluated on the stacked-ensemble model. Details of sets for hyperparameter tuning are summarized in the Supplemental Materials (Table S1).

Given the limited sample size, model training and evaluation employed a rigorous 5-fold cross-validation approach. The dataset was partitioned into 5 distinct subsets. In each fold, 4 subsets were used for training the ensemble model (including base learners and stackers), and the remaining subset was held out for testing. This process was repeated 5 times, ensuring each subset served as the test set exactly once. Crucially, the same data partitioning was maintained across all algorithm comparisons to ensure fairness.

### MO integration for pCR prediction

To develop the optimal pre-NAC model for predicting the primary endpoint, i.e., pCR, we established an MO model integrating the new RadS_ET_ and RadS_ITH_ signatures with clinical, pathological, and molecular variables derived from biopsy. This MO model incorporated patient baseline characteristics: age, tumor size, biopsy pathological grade, clinical tumor stage (T stage), nodal stage (N stage), ER/PR status, HER2 status, molecular classification (TNBC, TPBC, HER2-positive, luminal A, or luminal B), Ki-67 index, and NAC regimen (drugs and cycles). Similar to the radiomics analysis, all omics biomarkers were integrated using auto-stepwise stacked-ensemble learning. SHAP analysis was employed to quantify and visualize the contribution of each omics feature, enhancing the interpretability of the MO model for pCR prediction. Figure 1C presents the flowchart for the MO analysis of NAC response.

Then, we assessed the ability of MO integration to predict post-NAC response status according to RECIST version 1.1. Responders were defined as patients achieving a complete response or partial response, while nonresponders were those with stable disease or progressive disease at the post-NAC RECIST assessment.

Following surgical intervention, patients underwent consistent follow-up at 3- to 6-month intervals, based on serum and/or imaging examinations per standard oncological practice. Follow-up data were obtained from hospital records and/or telephone interviews. The survival endpoint, i.e., DFS, was defined as the time interval from the date of surgery to the detection of disease progression or recurrence. For patients who died from causes unrelated to BC or did not experience disease progression, follow-up time was censored at the date of last contact or death.

### Statistical analysis

Continuous variables (e.g., lesion size and imaging measurements) are presented as mean ± standard deviation. Categorical variables are presented as frequencies and proportions. Differences in clinical, pathological, and imaging characteristics between the pCR and non-pCR groups were compared using Student’s *t* test or the Mann–Whitney *U* test, as appropriate based on data distribution. The diagnostic efficacy of the predictive model was evaluated by calculating the area under the receiver operating characteristic curve (AUC-ROC). The optimal diagnostic threshold for calculating sensitivity, specificity, and accuracy was determined using the maximum Youden index. Seventeen MO features were prioritized using stepwise feature selection based on their relevance to pCR, aiming to balance model interpretability with predictive power. Furthermore, survival outcome was plotted via a Kaplan–Meier curve. Statistical analysis was performed with MedCalc software (version 15.2), Stata (version 17.0), and Python (version 3.8). Statistical significance was set at *P* < 0.05.

## Ethical Approval

The research protocol was approved by the local institutional ethics review committee. In accordance with the nature of the study and the policy of anonymizing data, written informed consent was waived. All research involving human participants complied with the principles of the 1964 Declaration of Helsinki and its later amendments.

## Data Availability

The imaging studies and clinical data used for algorithm development are not publicly available, because they contain private patient health information. Interested users may request access to these data, where institutional approvals along with signed data use agreements and/or material transfer agreements may be needed/negotiated. Derived result data supporting the findings of this study are available upon reasonable requests.
