# Supplementary material for: Multiomics Machine Learning to Predict Neoadjuvant Chemotherapy Outcome and Relapse of Breast Cancer
Source: BME Front. 2026 Jan 27;7:0212. doi: 10.34133/bmef.0212 (PMC12835490; doi:10.34133/bmef.0212)
Supplement: Supplementary 1 — Text S1 Table S1 [file bmef.0212.f1.docx]

**Supplemental file**

**Text-E1**

**Radiomics features:**

1) First-order statistics describe the histogram of voxel intensity values within the ROI through commonly used and basic metrics.

Let:

$X$ be a set of $N_{P}$ voxels included in the ROI,

$P(i)$ be the first order histogram with $N_{g}$ discrete intensity levels, where $N_{g}$ is the number of non-zero bins, equally spaced from 0 with a width defined in the binWidth parameter,

$p(i)$ be the normalized first order histogram and equal to$\frac{P(i)}{N_{P}}$.

1. Energy:

$$energy=\sum_{i=1}^{N_{P}} {(X\left( i \right)+c)}^{2}$$

2. Total Energy

$$total energy=V_{voxel}\sum_{i=1}^{N_{P}} {(X\left( i \right)+c)}^{2}$$

3. Entropy:

$$entropy=-\sum_{i=1}^{N_{g}} p(i)\log_{2} (p\left( i \right)+\epsilon)$$

Here, $\epsilon$ is an arbitrarily small positive number (≈2·2×10^−16^)

4. Minimum:

$$minimum=min(X)$$

5. 10th Percentile

The 10th percentile of$X$, a robust alternative to the minimum gray-value.

6. 90th Percentile

The 90th percentile of$X$, a robust alternative to the maximum gray-value.

7. Maximum:
The maximum gray level intensity within the ROI.

$$maximum=max(X)$$

8. Mean:

The average gray level intensity within the ROI.

$$mean=\frac{1}{N_{P}}\sum_{i=1}^{N_{P}} X(i)$$

9. Median:
The median gray level intensity within the ROI.

10. Interquartile Range:

$$interquartile range=P_{75}-P_{25}$$

Here $P_{25}$ and $P_{75}$ are the 25th and 75th percentile of the image array, respectively.

11. Range:
The range of gray values in the ROI.

$$range=\max\left( X \right)-min(X)$$

12. Mean Absolute Deviation (MAD):

MAD is the mean distance of all intensity values from the Mean Value of the image array.

$$MAD=\frac{1}{N_{P}}\sum_{i=1}^{N_{P}} \left| X(i)-\bar{X} \right|$$

Where $\bar{X}$ is the mean of $X$

13. Robust Mean Absolute Deviation (rMAD):

$$rMAD=\frac{1}{N_{10-90}}\sum_{i=1}^{N_{10-90}} \left| X_{10-90}\left( i \right)- \bar{X}_{10-90} \right|$$

rMAD is the mean distance of all intensity values from the Mean Value calculated on the subset of image array with gray levels in between, or equal to the 10th and 90th percentile.

14. Root Mean Square (RMS):

$$RMS=\sqrt{\frac{\sum_{i=1}^{N_{P}} {(X\left( i \right)+c)}^{2}}{N_{P}}}$$

Here, $c$ is optional value, defined by voxelArrayShift, which shifts the intensities to prevent negative values in $X$. This ensures that voxels with the lowest gray values contribute the least to RMS, instead of voxels with gray level intensity closest to 0.

15. Standard Deviation:

$$standard deviation=\sqrt{\frac{1}{N_{P}}\sum_{i=1}^{N_{P}} {(X\left( i \right)-\bar{X})}^{2}}$$

Where$\bar{X}$ is the mean of $X$.

16. Skewness:

Skewness measures the asymmetry of the distribution of values about the Mean value.

$$skewness=\frac{\frac{1}{N_{P}}\sum_{i=1}^{N_{P}N} {(X\left( i \right)-\bar{X})}^{3}}{\left( \sqrt{\frac{1}{N_{P}}\sum_{i=1}^{N_{P}} {(X\left( i \right)-\bar{X})}^{2}} \right)^{3}}$$

where $\bar{X}$ is the mean of $X$.

17. Kurtosis:

$$kurtosis=\frac{\frac{1}{N_{P}}\sum_{i=1}^{N_{P}} {(X\left( i \right)-\bar{X})}^{4}}{\left( \sqrt{\frac{1}{N_{P}}\sum_{i=1}^{N_{P}} \left( X\left( i \right)-\bar{X} \right)^{2}} \right)^{2}}$$

Where $\bar{X}$ is the mean of $X$.

18. Variance:

$$variance=\frac{1}{N_{P}}\sum_{i=1}^{N_{P}} {(X\left( i \right)-\bar{X})}^{2}$$

19. Uniformity:

$$uniformity=\sum_{i=1}^{N_{g}} {P(i)}^{2}$$

2) Shape features describe the three-dimensional size and shape of the ROI. These features are independent from the gray level intensity distribution in the ROI and are therefore only calculated on the non-derived image and mask.

Let:

$V$the volume of the ROI in mm3,

$A$ the surface area of the ROI in mm2.

1. Volume:

$$V=\sum_{i=1}^{N} V_{i}$$

The volume of the ROI $V$ is approximated by multiplying the number of voxels in the ROI by the volume of a single voxel $V_{i}$.

2. Surface Area:

$$A=\sum_{i=1}^{N} \frac{1}{2}\left| a_{i}b_{i}\times a_{i}c_{i} \right|$$

Where: $N$ is the number of triangles forming the surface mesh of the ROI $a_{i}b_{i}$ and $a_{i}c_{i}$ are the edges of the ithith triangle formed by points $a_{i}$, $b_{i}$ and $c_{i}$.

3. Surface to Volume Ratio:

$$surface to volume ratio=\frac{A}{V}$$

4. Sphericity:

Sphericity is a measure of how much the volume resembles a sphere.

$$sphericity=\frac{{(36\pi V^{2})}^{\frac{2}{3}}}{A}$$

5. Compactness 1:

Compactness is a measure of how much the volume resembles a sphere [1].

$$compactness 1=\frac{V}{\sqrt{\pi}A^{\frac{2}{3}}}$$

6. Compactness 2:

$$compactness 2=36\pi\frac{V^{2}}{A^{3}}$$

7. Spherical Disproportion:

Spherical disproportion is a measure of how much the volume resembles a sphere.

$$spherical disproportion=\frac{A}{4\pi R^{2}}$$

Where $A$ is the surface area and $R$is the radius of a sphere with the same volume as the tumor.

8. Maximum 3D diameter:

Maximum 3D diameter is defined as the largest pairwise Euclidean distance between surface voxels in the ROI.

9. Maximum 2D diameter (Slice):

Maximum 2D diameter (Slice) is defined as the largest pairwise Euclidean distance between tumor surface voxels in the row-column (generally the axial) plane.

10. Maximum 2D diameter (Row):

Maximum 2D diameter (Row) is defined as the largest pairwise Euclidean distance between tumor surface voxels in the column-slice (usually the sagittal) plane.

11. Maximum2D diameter (Column):

Maximum 2D diameter (Column) is defined as the largest pairwise Euclidean distance between tumor surface voxels in the row-slice (usually the coronal) plane.

12. Major Axis:

$$major axis=4\sqrt{\lambda_{\mathrm{major}}}$$

13. Minor Axis:

$$minor axis=4\sqrt{\lambda_{\mathrm{minor}}}$$

14. Least Axis:

$$least axis=4\sqrt{\lambda_{\mathrm{least}}}$$

15. Elongation:

$$elongation=\sqrt{\frac{\lambda_{\mathrm{minor}}}{\lambda_{\mathrm{major}}}}$$

Here, $\lambda_{\mathrm{major}}$and $\lambda_{\mathrm{minor}}$ are the lengths of the largest and second largest principal component axes.

16. Flatness

$$flatness=\sqrt{\frac{\lambda_{\mathrm{least}}}{\lambda_{\mathrm{major}}}}$$

Here, $\lambda_{\mathrm{major}}$and $\lambda_{\mathrm{least}}$ are the lengths of the largest and smallest principal component axes.

3) Statistics-based textural features describe patterns or the spatial distribution of voxel intensities, which were calculated from respectively gray-level co-occurrence matrix (GLCM), gray-level run length matrix (GLRLM), and gray-level size zone matrix (GLSZM) features.

Gray Level Co-occurrence Matrix (GLCM) Features

A normalized GLCM is defined as $P(i,j;\delta,\alpha)$, a matrix with size $N_{g}\times N_{g}$ describing the second-order joint probability function of an image, where the ${(i,j)}^{\mathrm{th}}$ element represents the number of times the combination of intensity levels $i$ and $j$ occur in two pixels in the image, that are separated by a distance of $\delta$ pixels in direction $\alpha$. The distance δ from the center voxel is defined as the distance according to the infinity norm. For δ=1, this results in 2 neighbors for each of 13 angles in 3D (26-connectivity).

Let:

$\epsilon$ be an arbitrarily small positive number (≈2·2×10^−16^)

$P\left( i,j \right)$ be the co-occurence matrix for an arbitrary$\delta$ and $\alpha$, ,

$p(i,j)$ be the normalized co-occurence matrix and equal to$\frac{P(i,j)}{\sum P(i,j)}$,

$N_{g}$ be the number of discrete intensity levels in the image,

$p_{x}\left( i \right)=\sum_{j=1}^{N_{g}} P(i,j)$ be the marginal row probabilities,

$p_{y}\left( i \right)=\sum_{i=1}^{N_{g}} P(i,j)$ be the marginal column probabilities,

$\mu_{x}$ be the mean gray level intensity of $p_{x}$,

$\mu_{y}$ be the mean gray level intensity of $p_{y}$,

$\sigma_{x}$ be the standard deviation of $p_{x}$,

$\sigma_{y}$ be the standard deviation of $p_{y}$,

$p_{x+y}\left( k \right)=\sum_{i=1}^{N_{g}} \sum_{j=1}^{N_{g}} P\left( i,j \right)$, where $i+j=k$, and $k=2,3,\ldots,2N_{g}$,

$p_{x-y}\left( k \right)=\sum_{i=1}^{N_{g}} \sum_{j=1}^{N_{g}} P\left( i,j \right)$, where $\left| i-j \right|=k$, and $k=0,1,\ldots,N_{g}-1$,

$HX=-\sum_{i=1}^{N_{g}} p_{x}(i)\log_{2} \left[ p_{x}(i)+\epsilon\right]$ be the entropy of $p_{x}$,

$HY=-\sum_{i=1}^{N_{g}} p_{y}(i)\log_{2} \left[ p_{y}(i)+\epsilon\right]$ be the entropy of $p_{y}$,

$HXY=-\sum_{i=1}^{N_{g}} \sum_{j=1}^{N_{g}} P\left( i,j \right)\log_{2} \left[ P\left( i,j \right)+\epsilon\right]$ be the entropy of $P(i,j)$,

$HXY1=-\sum_{i=1}^{N_{g}} \sum_{j=1}^{N_{g}} P\left( i,j \right)\log_{2}(p_{x}\left( i \right)p_{y}\left( j \right)+\epsilon)$,

$$HXY2=-\sum_{i=1}^{N_{g}} \sum_{j=1}^{N_{g}} p_{x}\left( i \right)p_{y}(j)\log_{2}(p_{x}\left( i \right)p_{y}\left( j \right)+\epsilon)$$

1. Autocorrelation

$$autocorrelation=\sum_{i=1}^{N_{g}} \sum_{j=1}^{N_{g}} ijp(i,j)$$

2. Joint Average

$$joint average=\mu x=\sum_{i=1}^{N_{g}} \sum_{j=1}^{N_{g}} p\left( i,j \right)i$$

3. Cluster Prominence:

$$cluster prominence=\sum_{i=1}^{N_{g}} \sum_{j=1}^{N_{g}} {(i+j-\mu_{x}-\mu_{y})}^{4}p(i,j)$$

4. Cluster Shade:

$$cluster shade=\sum_{i=1}^{N_{g}} \sum_{j=1}^{N_{g}} {(i+j-\mu_{x}-\mu_{y})}^{3}p(i,j)$$

5. Cluster Tendency:

$$cluster tendency=\sum_{i=1}^{N_{g}} \sum_{j=1}^{N_{g}} {(i+j-\mu_{x}-\mu_{y})}^{2}p(i,j)$$

6. Contrast:

$$contrast=\sum_{i=1}^{N_{g}} \sum_{j=1}^{N_{g}} {(i-j)}^{2}p(i,j)$$

7. Correlation:

$$correlation=\frac{\sum_{i=1}^{N_{g}} \sum_{j=1}^{N_{g}} ijp\left( i,j \right)-\mu_{x}\mu_{y}}{\sigma_{x}(i)\sigma_{y}(j)}$$

8. Difference Average:

$$difference average =\sum_{k=0}^{N_{g}-1} kp_{x-y}(k)$$

9. Difference Entropy:

$$difference entropy=\sum_{k=0}^{N_{g}-1} p_{x-y}(k)\log_{2} (p_{x-y}\left( k \right)+\epsilon)$$

10. Difference Variance:

$$difference variance=\sum_{k=0}^{N_{g}-1} {{(k-DA)}^{2}p}_{x-y}(k)$$

11. Dissimilarity:

$$dissimilarity=\sum_{i=1}^{N_{g}} \sum_{j=1}^{N_{g}} \left| i-j \right|p(i,j)$$

12. Joint Energy:

$$joint energy=\sum_{i=1}^{N_{g}} \sum_{j=1}^{N_{g}} {(p(i,j))}^{2}$$

13. Joint Entropy:

$$joint entropy=-\sum_{i=1}^{N_{g}} \sum_{j=1}^{N_{g}} p\left( i,j \right)\log_{2} (p\left( i,j \right)+\epsilon)$$

14. Homogeneity 1:

$$homogeneity 1=\sum_{i=1}^{N_{g}} \sum_{j=1}^{N_{g}} \frac{p(i,j)}{1+\left| i-j \right|}$$

15. Homogeneity 2:

$$homogeneity 2=\sum_{i=1}^{N_{g}} \sum_{j=1}^{N_{g}} \frac{p(i,j)}{1+\left| i-j \right|^{2}}$$

16. Informal Measure of Correlation (IMC) 1:

$$IMC1=\frac{HXY-HXY1}{\text{max}\left\{ HX,HY \right\}}$$

17. Informal Measure of Correlation (IMC) 2:

$$IMC2=\sqrt{1-e^{-2(HXY2-HXY)}}$$

18. Inverse Difference Moment (IDM)

$$IDM=\sum_{i=1}^{N_{g}} \sum_{j=1}^{N_{g}} \frac{p(i,j)}{1+\left| i-j \right|^{2}}$$

19. Inverse Difference Moment Normalized (IDMN):

$$IDMN=\sum_{i=1}^{N_{g}} \sum_{j=1}^{N_{g}} \frac{p(i,j)}{1+\left( \frac{\left| i-j \right|^{2}}{{N_{g}}^{2}} \right)}$$

20. Inverse Difference (ID):

$$ID=\sum_{i=1}^{N_{g}} \sum_{j=1}^{N_{g}} \frac{p(i,j)}{1+\left| i-j \right|}$$

21. Inverse Difference Normalized (IDN):

$$IDN=\sum_{i=1}^{N_{g}} \sum_{j=1}^{N_{g}} \frac{p(i,j)}{1+\left( \frac{\left| i-j \right|}{N_{g}} \right)}$$

22. Inverse Variance:

$$\begin{matrix} inverse variance=\sum_{i=1}^{N_{g}} \sum_{j=1}^{N_{g}} \frac{p(i,j)}{\left| i-j \right|^{2}} & ,i\neq j \end{matrix}$$

23. Maximum Probability:

$$maximum probability=\text{max}(p(i,j))$$

24. Sum Average:

$$sum average=\sum_{k=2}^{2N_{g}} (kp_{x+y}(k))$$

25. Sum Entropy:

$$sum entropy=-\sum_{k=2}^{2N_{g}} p_{x+y}(k)\log_{2} (p_{x+y}(k)+\epsilon)$$

26. Sum of Squares:

$$sum squares=\sum_{i=1}^{N_{g}} \sum_{j=1}^{N_{g}} \left( i-\mu_{x} \right)^{2}p(i,j)$$

Gray Level Run Length Matrix (GLRLM) Features

A GLRLM quantifies gray level runs, which are defined as the length in number of pixels, of consecutive pixels that have the same gray level value. In a gray level run length matrix $P(i,j|\theta)$, the ${(i,j)}^{\mathrm{th}}$element describes the number of runs with gray level $i$ and length $j$ occur in the image along angle $\theta$.

Let:

$N_{g}$ be the number of discreet intensity values in the image,

$N_{r}$ be the number of discreet run lengths in the image,

$N_{p}$ be the number of voxels in the image,

$N_{z}(\theta)$ be the number of runs in the image along angle $\theta$, which is equal to $\sum_{i=1}^{N_{g}} \sum_{j=1}^{N_{r}} P(i,j|\theta)$, and ${1\leq N}_{z}(\theta)\leq N_{p}$,

$P(i,j|\theta)$ be the run length matrix for an arbitrary direction$\theta$,

$p(i,j|\theta)$ be the normalized run length matrix, defined as$p\left( i,j | \theta\right)=\frac{P(i,j|\theta)}{N_{z}(\theta)}$,

1. Short Run Emphasis (SRE):

$$SRE=\frac{\sum_{i=1}^{N_{g}} \sum_{j=1}^{N_{r}} \frac{P(i,j|\theta)}{j^{2}}}{N_{z}(\theta)}$$

2. Long Run Emphasis (LRE):

$$LRE=\frac{\sum_{i=1}^{N_{g}} \sum_{j=1}^{N_{r}} j^{2}P(i,j|\theta)}{N_{z}(\theta)}$$

3. Gray Level Non-Uniformity (GLN):

$$GLN=\frac{\sum_{i=1}^{N_{g}} {(\sum_{j=1}^{N_{r}} p(i,j|\theta))}^{2}}{N_{z}(\theta)}$$

4. Gray Level Non-Uniformity Normalized (GLNN):

$$GLNN=\frac{\sum_{i=1}^{N_{g}} {(\sum_{j=1}^{N_{r}} p(i,j|\theta))}^{2}}{{N_{z}(\theta)}^{2}}$$

5. Run Length Non-Uniformity (RLN):

$$RLN=\frac{\sum_{j=1}^{N_{r}} {(\sum_{i=1}^{N_{g}} P(i,j|\theta))}^{2}}{N_{z}(\theta)}$$

6. Run Length Non-Uniformity Normalized (RLNN):

$$RLNN=\frac{\sum_{j=1}^{N_{r}} {(\sum_{i=1}^{N_{g}} P(i,j|\theta))}^{2}}{{N_{z}(\theta)}^{2}}$$

7. Run Percentage (RP):

$$RP=\sum_{i=1}^{N_{g}} \sum_{j=1}^{N_{r}} \frac{P(i,j|\theta)}{N_{p}}$$

8. Gray Level Variance (GLV):

$$GLV=\sum_{i=1}^{N_{g}} \sum_{j=1}^{N_{r}} p(i,j|\theta){(i-\mu)}^{2}$$

Here,$\mu=\sum_{i=1}^{N_{g}} \sum_{j=1}^{N_{r}} p(i,j|\theta)i$

9. Run Variance (RV):

$$RV=\sum_{i=1}^{N_{g}} \sum_{j=1}^{N_{r}} p(i,j|\theta){(j-\mu)}^{2}$$

Here,$\mu=\sum_{i=1}^{N_{g}} \sum_{j=1}^{N_{r}} p(i,j|\theta)j$

10. Run Entropy (RE):

$$RE=\sum_{i=1}^{N_{g}} \sum_{j=1}^{N_{r}} p(i,j|\theta)\log_{2} (p(i,j|\theta)+\epsilon)$$

Here, $\epsilon$ is an arbitrarily small positive number (≈2.2×10−16)

11. Low Gray Level Run Emphasis (LGLRE):

$$LGLRE=\frac{\sum_{i=1}^{N_{g}} \sum_{j=1}^{N_{r}} \frac{P(i,j|\theta)}{i^{2}}}{N_{z}(\theta)}$$

12. High Gray Level Run Emphasis (HGLRE):

$$HGLRE=\frac{\sum_{i=1}^{N_{g}} \sum_{j=1}^{N_{r}} i^{2}P(i,j|\theta)}{N_{z}(\theta)}$$

13. Short Run Low Gray Level Emphasis (SRLGLE):

$$SRLGLE=\frac{\sum_{i=1}^{N_{g}} \sum_{j=1}^{N_{r}} \frac{P(i,j|\theta)}{i^{2}j^{2}}}{N_{z}(\theta)}$$

14. Short Run High Gray Level Emphasis (SRHGLE):

$$SRHGLE=\frac{\sum_{i=1}^{N_{g}} \sum_{j=1}^{N_{r}} \frac{P(i,j|\theta)i^{2}}{j^{2}}}{N_{z}(\theta)}$$

15. Long Run Low Gray Level Emphasis (LRLGLE):

$$LRLGLE=\frac{\sum_{i=1}^{N_{g}} \sum_{j=1}^{N_{r}} \frac{P(i,j|\theta)j^{2}}{i^{2}}}{N_{z}(\theta)}$$

16. Long Run High Gray Level Emphasis (LRHGLE):

$$LRHGLE=\frac{\sum_{i=1}^{N_{g}} \sum_{j=1}^{N_{r}} P(i,j|\theta)i^{2}j^{2}}{N_{z}(\theta)}$$

Gray Level Size Zone Matrix (GLSZM) Features

A GLSZM describes the amount of homogeneous connected areas within the volume, of a certain size and intensity, thereby describing tumor heterogeneity at a regional scale [2]. A voxel is considered connected if the distance is 1 according to the infinity norm (26-connected region in 3D). In a GLSZM $P(i,j)$, the ${(i,j)}^{\mathrm{th}}$element equals the number of zones with gray level $i$ and size $j$ appear in image. Contrary to GLCM and GLRLM, the GLSZM is rotation independent, with only one matrix calculated for all directions in the ROI. The mathematical formulas that define the GLSZM features correspond to the definitions of features extracted from the GLRLM.

Let:

$N_{g}$ be the number of discreet intensity values in the image,

$N_{s}$ be the number of discreet zone sizes in the image,

$N_{p}$ be the number of voxels in the image,

$N_{z}$ be the number of zones in the ROI, which is equal to $\sum_{i=1}^{N_{g}} \sum_{j=1}^{N_{s}} P\left( i,j \right)$, and ${1\leq N}_{z}N_{z}\leq N_{p}$

$P(i,j)$ be the size zone matrix,

$p(i,j)$ be the normalized size zone matrix, defined as $p\left( i,j \right)= \frac{P(i,j)}{N_{z}}$.

1. Small Area Emphasis (SAE):

$$SAE=\frac{\sum_{i=1}^{N_{g}} \sum_{j=1}^{N_{s}} \frac{P(i,j)}{j^{2}}}{N_{z}}$$

2. Large Area Emphasis (LAE):

$$LAE=\frac{\sum_{i=1}^{N_{g}} \sum_{j=1}^{N_{s}} P(i,j)j^{2}}{N_{z}}$$

3. Gray Level Non-Uniformity (GLN):

$${GLN=\frac{\sum_{i=1}^{N_{g}} (\sum_{j=1}^{N_{s}} P(i,j))}{N_{z}}}^{2}$$

4. Gray Level Non-Uniformity Normalized (GLNN):

$${GLNN=\frac{\sum_{i=1}^{N_{g}} (\sum_{j=1}^{N_{s}} P(i,j))}{{N_{z}}^{2}}}^{2}$$

5. Size-Zone Non-Uniformity (SZN):

$${SZN=\frac{\sum_{j=1}^{N_{s}} (\sum_{i=1}^{N_{g}} P(i,j))}{N_{z}}}^{2}$$

6. Size-Zone Non-Uniformity Normalized (SZNN):

$${SZNN=\frac{\sum_{j=1}^{N_{s}} (\sum_{i=1}^{N_{g}} P(i,j))}{{N_{z}}^{2}}}^{2}$$

7. Zone Percentage (ZP):

$$ZP=\frac{N_{z}}{N_{p}}$$

8. Gray Level Variance (GLV):

$$GLV=\sum_{i=1}^{N_{g}} \sum_{j=1}^{N_{s}} p(i,j){(i-\mu)}^{2}$$

Here,$\mu=\sum_{i=1}^{N_{g}} \sum_{j=1}^{N_{s}} p(i,j)i$

9. Zone Variance (ZV):

$$ZV=\sum_{i=1}^{N_{g}} \sum_{j=1}^{N_{s}} p(i,j){(j-\mu)}^{2}$$

Here,$\mu=\sum_{i=1}^{N_{g}} \sum_{j=1}^{N_{s}} p(i,j)j$

10. Zone Entropy (ZE):

ZE measures the uncertainty/randomness in the distribution of zone sizes and gray levels. A higher value indicates more heterogeneneity in the texture patterns.

$$ZE=\sum_{i=1}^{N_{g}} \sum_{j=1}^{N_{s}} p(i,j)\log_{2} \left[ p(i,j)+\epsilon\right]$$

Here, $\epsilon$ is an arbitrarily small positive number (≈2·2×10^−16)^

11. Low Gray Level Zone Emphasis (LGLZE):

$$LGLZE=\frac{\sum_{i=1}^{N_{g}} \sum_{j=1}^{N_{s}} \frac{P(i,j)}{i^{2}}}{N_{z}}$$

12. High Gray Level Zone Emphasis (HGLZE):

$$HGLZE=\frac{\sum_{i=1}^{N_{g}} \sum_{j=1}^{N_{s}} i^{2}P(i,j)}{N_{z}}$$

13. Small Area Low Gray Level Emphasis (SALGLE):

$$SALGLE=\frac{\sum_{i=1}^{N_{g}} \sum_{j=1}^{N_{s}} \frac{P(i,j)}{i^{2}j^{2}}}{N_{z}}$$

14. Small Area High Gray Level Emphasis (SAHGLE):

$$SAHGLE=\frac{\sum_{i=1}^{N_{g}} \sum_{j=1}^{N_{s}} \frac{P(i,j)i^{2}}{j^{2}}}{N_{z}}$$

15. Large Area Low Gray Level Emphasis (LALGLE):

$$LALGLE=\frac{\sum_{i=1}^{N_{g}} \sum_{j=1}^{N_{s}} \frac{P(i,j)j^{2}}{i^{2}}}{N_{z}}$$

16. Large Area High Gray Level Emphasis (LAHGLE):

$$HGLZE=\frac{\sum_{i=1}^{N_{g}} \sum_{j=1}^{N_{s}} i^{2}j^{2}P(i,j)}{N_{z}}$$

| **Table E1**  **Hyper-parameter space search configuration of 7 base learners.** | |
| --- | --- |
| Learner | Hyper-parameter set configuration |
| NNs | Hidden layers (100, 100); Activation (ReLu);  Solver (Adam); Regularization (0.01 to 1, step 0.01) |
| XGBoost | Tree (10 to 400); Learning rate (0.1 to 1, step 0.1);  Regularization (0.01 to 1, step 0.01); Depth (3-15, step 1) |
| CatBoost | Tree (10 to 400); Learning rate (0.1 to 1, step 0.1);  Depth (3-15, step 1) |
| NB | - |
| XGB-RF | Tree (10 to 400); Learning rate (0.1 to 1, step 0.1);  Regularization (0.01 to 1, step 0.01); Depth (3-15, step 1) |
| LR | Lasso regression;  Cost (0.1 to 1, step 0.1) |
| RFs | Tree (10 to 400); Learning rate (0.1 to 1, step 0.1);  Regularization (0.01 to 1, step 0.01); Depth (3-15, step 1) |
| NNs = Neural Networks; XGBoost = Extreme Gradient Boosting; CatBoost = gradient boosting with categorical features support; NB = Naïve Bayes; LR = Logistic Regression; RFs = Random Forests. | |

**Reference**

1. Lambin P, Leijenaar RTH, Deist TM, et al. Radiomics: the bridge between medical imaging and personalized medicine. Nat Rev Clin Oncol 2017; 14:749-762. doi: 10.1158/0008-5472

2. Van Griethuysen JJM, Fedorov A, Parmar C, et al. Computational Radiomics System to Decode the Radiographic Phenotype. Cancer Res 2017; 77:e104-e107. doi: 10.1158/0008-5472.
